# Endothelial reconstitution by CD34+ progenitors derived from baboon embryonic stem cells

**DOI:** 10.1111/jcmm.12002

**Published:** 2013-01-10

**Authors:** Qiang Shi, Gerald Schatten, Vida Hodara, Calvin Simerly, John L VandeBerg

**Affiliations:** a Southwest National Primate Research Center, Texas Biomedical Research InstituteSan Antonio, TX, USA; b Pittsburgh Development Center, University of Pittsburgh School of MedicinePittsburgh, PA, USA

**Keywords:** Embryonic stem cells, Endothelial differentiation, Specification and maturation, Stem cell therapy, Non-human primate model

## Abstract

In this study, we used a large non-human primate model, the baboon, to establish a step-wise protocol to generate CD34+ endothelial progenitor cells (EPCs) from embryonic stem cells (ESCs) and to demonstrate their reparative effects. Baboon ESCs were sequentially differentiated from embryoid body cultures for 9 days and then were specified into EPCs by culturing them in monolayer for 12 days. The resulting EPCs expressed CD34, CXCR4 and UEA-1, but neither CD31 nor CD117. The EPCs were able to form intact lumen structures when seeded on Matrigel, took up Dil-LDL, and responded to TNF-α. Angioblasts specified in EGM-2 medium and ECGS medium had 6.41 ± 1.16% (*n* = 3) and 9.32 ± 3.73% CD34+ cells (*n* = 3). The efficiency of generating CD34+ EPCs did not differ significantly from ECGS to EGM-2 culture media, however, angioblasts specified in ECGS medium expressed a higher percentage of CD34+/CXCR4+ cells (3.49 ± 1.32%, *n* = 3) than those specified in EGM-2 medium (0.49 ± 0.52%, *n* = 3). To observe their reparative capacity, we purified CD34+ progenitors after specification by EGM-2 medium; inoculated fluorescently labelled CD34+ EPCs into an arterial segment denuded of endothelium in an *ex vivo* system. After 14 days of *ex vivo* culture, the grafted cells had attached and integrated to the denuded surface; in addition, they had matured further and expressed terminally differentiated endothelial markers including CD31 and CD146. In conclusion, we have proved that specified CD34+ EPCs are promising therapeutic agents for repairing damaged vasculature.

## Introduction

Numerous studies over the last decade have demonstrated that adult tissues, including bone marrow [1], adipose tissue [2, 3] and the vascular wall [4, 5], contain primitive stem cells that have the potential to differentiate into endothelial cells in response to vascular injury. These cells are collectively known as endothelial progenitor cells (EPCs). Post-natal endothelial growth and remodelling depend not only on the proliferation and sprouting of pre-existing endothelial cells but also on the differentiation of these adult stem cells hibernating in special niches in adult tissues. Strong evidence supports the notion that EPCs are important cellular sources for vascular repair because they can mobilize into the circulation, incorporate into injured vessels and facilitate neovascularization processes [6–9]. Therefore, once grown in sufficient number *in vitro*, EPCs could be used as a new agent in therapeutic application [10–12].

Pluripotent stem cells of embryonic origin offer the possibility of a renewable and inexhaustible source of therapeutic cells, but optimal clinical benefit cannot be achieved without a good understanding of what types of cells should be transplanted. Although functional progenitor cells derived from stem cells have been widely investigated in various models, the lack of direct evidence to prove their therapeutic efficacy is the greatest challenge in moving stem cell therapy for treating vascular diseases into the clinical setting. There is an increasing need for an animal model that yields optimal results that are directly relevant to humans because dramatically different mechanisms regulate stem cell differentiation among species, and the modes of stem cell delivery (dosage, time-points, methods) have a profound effect on the efficacy of treatment [10, 13, 14]. Attempts to apply methods developed in small animal models to human beings are usually problematic.

The baboon has an advantage over other models because its physiological characteristics are highly similar to those of human beings [15–17]. In this study, we demonstrate that progenitor cells derived from baboon embryonic stem cells (ESCs) can home, proliferate and differentiate into mature cells when they are transplanted into damaged tissues. Acknowledging that ESC differentiation is difficult to direct [18, 19], we devised a strategy to recapitulate the *in vivo* developmental stages of ESCs under *in vitro* conditions, thus to derive EPCs from pluripotent stem cells into mesodermal haemangioblasts [20]. Following that process, we specified derivatives into highly restricted cells of endothelial lineage. This strategy allows us to harvest proliferative progenitors with decreased pluripotency but directed differentiation. Our studies show that CD34+ EPCs generated under this protocol exhibit a profound ability in an *ex vivo* model system to re-endothelialize denuded arteries of baboons within 2 weeks, and that the progenitor cells gain further maturation towards functional competence after they have been transplanted into the arteries.

## Materials and methods

### Cell culture and *in vitro* derivation

**Fig. 1:**
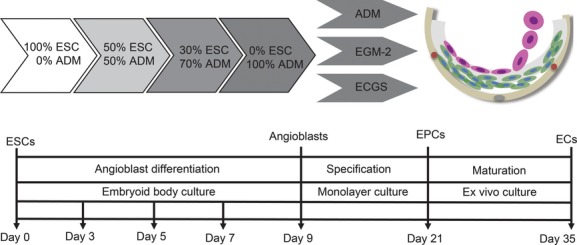
Experimental design for generating and identifying therapeutic EPCs from ESCs. EPCs are generated from ESCs *via* angioblast formation from EBs suspended in ESC/ADM media with varying ratios for indicated times, following by specification in monolayer culture on collagen-coated plates under three different media to form functional EPCs. Progenitors are matured on a microenvironment provided by injured vascular surface. Therapeutic effects are evaluated by transplanting EPCs onto a denuded vascular surface, which provides a suitable microenvironment for EPCs to mature towards functional competence.

The BAB15 baboon ESC lines at passage 39 were obtained from the Pittsburgh Development Center, University of Pittsburgh School of Medicine; they were cultured according to the published method and used within no more than 10 passages. We confirmed the cellular pluripotency by growth behaviour; positive immunostaining for OCT-4, NANOG and SSEA-4; and histochemical staining for ALP [21]. Pooled colonies of high-proliferative-potential endothelial colony-forming cells (HPP-ECFCs), which were considered tissue-resident EPCs, were isolated and cultured according to the method described previously [22]. We modified our protocol for differentiating endothelial progenitors from ESCs based on reported methods [23–26] and developed a step-wise differentiation protocol (Fig. 1). In the first stage, we aimed at differentiating angioblasts from ESCs in three-dimensional embryoid body (EB) culture. We derived angioblasts from ESCs in three-dimensional EB culture in angioblast differentiation medium (ADM). We used AggreWell plates to form uniform EBs containing about 5000–10,000 cells and cultured them in ESC medium for 3 days. The ADM consisted of ESC medium supplemented with a cocktail of 0.5 ng/ml BMP-4, 5 ng/ml basic FGF, 10 ng/ml VEGF, 5 ng/ml stem cell factor, 5 ng/ml thrombopoietin and 10 ng/ml FLT-3 ligand. Subsequently, we added ADM to EB cultures at gradually increased ratios at the time-points indicated in Figure 1. At the end of day nine, the EBs were transferred onto collagen-coated plates (BD Biosciences, San Jose, CA, USA) for monolayer culture [21]. At this stage, we specified angioblast preparations and generated endothelial progenitors. Two specifying media were used: (1) EGM-2 medium containing EGF, hydrocortisone, VEGF, FGF-B, R3-IGF-1, ascorbic acid, heparin, gentamicin, amphotericin-B, and FCS (Lonza, detailed concentrations are provided at http://www.lonza.com), and (2) ECGS medium with endothelial growth factors from bovine pituitary extracts (Sigma-Aldrich, St. Louis, MO, USA) [27]. At the same time, some cells were allowed to continue growing in ADM as a reference control. After 12 days, endothelial lineage progenitor cells were harvested by enzymatic digestion for various tests. To determine the average cell number per colony, we fixed the cells grown on 24-well plate and stained the cells with thiazine dye (HEMA-Diff kit, StatLab, TX, USA). We counted the stained nuclei of each cell with the Objective Micrometer (Meiji Techno America, San Jose, CA, USA) under the microscope to achieve the accurate cell count in colonies.

### Flow cytometry

We prepared single-cell suspensions from monolayer cultures by trypsin digestion for 10 min. and conducted flow cytometry [9]. Undifferentiated or adult endothelial cells served as controls for monitoring endothelial lineage differentiation. Table 1 lists vendors, clones and antibody dilutions. Flow cytometry was performed on a CyAn (DAKO; DAKO North America Inc., Carpinteria, CA, USA). All flow cytometry procedures were repeated at least three times.

**Table 1:** Antibodies used for flow cytometry and immunofluorescence

Antibodies	Vendors and Catalogue No.	Clone	Amount or Dilution
CD34-PE	BD Biosciences #550619	563	10 μl/10^6^ cells
CD31-FITC	BD Biosciences #557508	WM59	5 μl/10^6^ cells
CD117-APC	BD Biosciences #550412	YB5.B8	5 μl/10^6^ cells
CD45-PerCP	BD Biosciences #558411	DO58-1283	5 μl/10^6^ cells
CD146-FITC	R&D Systems FAB932F	12808	10 μl/10^6^ cells
CXCR4-APC	BioLegend #306510	12G5	5 μl/10^6^ cells
UEA-FITC	Sigma #9006		3 μl/10^6^ cells
VEGFR3-PE	R&D Systems #FAB3492P	54733	5 μl/10^6^ cells
CD54-APC	BD Biosciences #559771	HA58	10 μl/10^6^ cells
CD62E-FITC	R&D Systems # BBA21	BB1G-E5	10 μl/10^6^ cells
CD106-PE	US Biological #C2446-71E	5K26T	10 μl/10^6^ cells

### CD34+ EPC purification

CD34+ EPCs derived from angioblasts were purified by magnetic bead cell sorting techniques (StemCell Technology) as described in our previous publication [9]. A cell suspension containing 0.5–1.0 million cells was blocked by 5 μl FcR blocker. Anti-CD34 antibody conjugated with PE (BD Biosciences), a PE selection reagent and magnetic nanoparticles included in the kit were added according to the manufacturer's instructions. The purified cells were then washed twice in 2% FCS PBS and resuspended in the medium. Cell purities ranged from 85% to 90% after two rounds of purification.

### Dil-LDL uptake

Cells were incubated in EGM-2 medium containing 10 mg/ml Dil-LDL (BTI) without supplementing serum but with 5% calf albumin (Sigma-Aldrich) at 37°C for 4 hrs and then fixed in 2% paraformaldehyde (PFA) solution for 20 min. at room temperature [9]. Images were viewed with a Nikon Eclipse E800 fluorescence microscope (Nikon Instruments Inc., Melville, NY, USA).

### Tube formation by Matrigel

Matrigel (BD Biosciences) was thawed overnight at 4°C, and then 500 μl of the gel solution was added to each well of a pre-chilled 12-well sterile plate. Cell suspension (500 μl containing 10^5^ cells) was added to each well and the cells were incubated at 37°C in a 5% CO_2_ atmosphere. The formation of tube or lumen structures was observed and assessed after incubation for various time-points.

### Long-term tracing of labelled cells

To trace the transplanted cells in *ex vivo* culture, we labelled the cells with fluorescent CellTracker Probe, CMRA (Invitrogen, Grand Island, NY, USA). We replaced the culture medium with fresh medium containing 10 μM of CMRA and incubated the cells for 45 min. before transplanting them into denuded arteries, which in turn were placed in the *ex vivo* bioreactor.

### Construction and use of *ex vivo* bioreactor

The bioreactor has two parts: a rotary cylinder with four stainless steel tubes on both sides, and a laminar perfusion system driven by a digital peristaltic pump. A segment of baboon femoral artery 5–7 cm long and without any branches or holes on the wall was treated with 0.05% trypsin together with 0.1% collagenase and 0.1% DNase I for 30 min. at 37°C to remove the endothelium. After thoroughly washing the segment with medium to detach all endothelial cells, we seeded the transplanted cells onto the lumen of the denuded artery and used plastic tubing to ligate the two ends of the vessel segment to the steel tubes in the bioreactor to construct a circuit. Endothelial culture medium (EGM-2, Lonza, Clonetics, Walkersville, MD, USA) in a reservoir was perfused through the lumen of the vessel. The whole vessel was submerged in 10% FBS F-12K medium and placed in an incubator. In most instances, two arterial segments were harvested from the same animal and denuded under the same conditions, then cells were labelled using CellTracker as stated above. The treatment group received a cell suspension (2–5 × 10^5^) of ESC-derived progenitors by injection; the control group received same amount medium without cells by injecting into the second denuded arterial segment from the same baboon.

### Cellular response to TNF-α

When specified EPCs grown in monolayer reached 80–90% confluence, we replaced the medium with new medium containing 10 ng/ml TNF-α (Sigma-Aldrich). Then, after 4 hrs, we determined their CD62E (E-selectin), CD54 and CD106 expression by flow cytometry [28], detailed information about these antibodies are listed in Table 1.

### Immunofluorescence and whole-mount immunostaining

Arterial tissues with and without cell transplantation were embedded in OCT in a cryomold and overlaid with OCT. The samples were quickly frozen on dry ice; we cut sections of 5–10 μm on a superfrost slide and fixed them in 2% PFA at room temperature for 20 min. We processed the sections immediately with immunofluorescence staining as described above.

### Statistical analysis

Data are expressed as mean ± SD. Student *t*-tests were used to perform statistical analysis; *P* < 0.05 was considered as significant.

## Results

We have been suggested that the generation of EPCs would be more efficient if we could recapitulate the formation of EPCs *in vivo*, therefore, we modified previous methods [25, 29–32] and designed a step-wise derivation protocol to generate EPCs from ESCs in a space- and time-dependent manner (Fig. 1). The modifications included a transition from 3D culture (EB) to 2D culture (monolayer), as well as alterations in culture medium components that drive cellular differentiation and specification. Our results showed that differentiated ESCs gained fully developed functionalities specific to the mature endothelium.

### Effects of specification on ESC-derived cultures

**Fig. 2:**
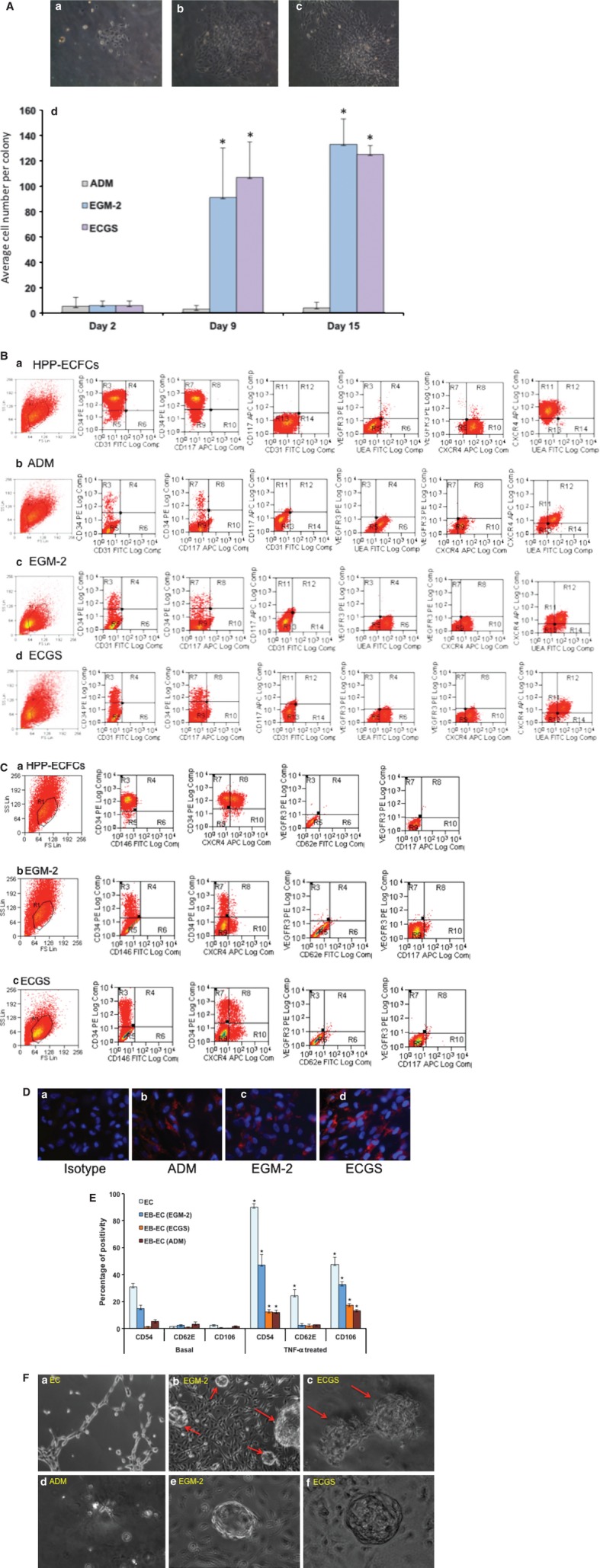
Specification of angioblasts induced by endothelial growth factors. (**A**) Proliferative potential of EPCs derived from ESCs. Morphologic features of angioblast cultures under EGM-2 differentiation are shown at day two (a), day nine (b) and day 15 (c). Panel d indicates the average cell number per colony during the culture. Significant differences existed between ADM and EGM-2 or ECGS; results are expressed as mean ± SD, *t*-test, *n* = 20. (**B**) Antigenic expression of angioblasts specified by different culture media. Flow cytometry results of specified EPCs together with HPP-ECFCs are presented. Single-cell suspensions were stained with the indicated antibody combination; all cells were gated as CD45−. Isotype-matched control antibody staining was performed and used to process the data. (**C**) Comparison of EGM-2 and ECGS in angioblast specification. Antigenic expression pattern by flow cytometry reveals the differences of angioblast specification under EGM-2 and ECGS, using HPP-ECFCs as a reference. (**D**) LDL uptake by angioblasts specified by three media. ESC-derived cells were grown in ADM (b), EGM-2 (c) and ECGS (d); and incubated with Dil-LDL to measure their uptake ability. Dil-LDL is labelled in red. Nuclei are stained blue with DAPI 400×. (**E**) Cellular reactivity to 10 ng/ml TNF-α. Specified EPCs under EGM-2 (dark blue), ECGS (orange), ADM (maroon), or control EC (light blue), were cultured in monolayer and treated with TNF-α for 4 hrs; corresponding controls were treated with an equal amount of PBS. Flow cytometry to detect CD54, CD62E and CD106 was conducted. Percentage positivity is shown. Results were calculated from three experiments and expressed as mean ± SD. ★ indicates significant difference between TNF-α-treated cells and corresponding controls (*P* < 0.05). (**F**) Vascular-like and microvessel formation on Matrigel. (a) endothelial cells isolated from blood vessels; (b and e) specified EPCs from EGM-2; (c and f) specified EPCs from ECGS; (d) specified EPCs from ADM. a–d, 100×; e–f, 400×.

As demonstrated previously, our angioblast differentiation protocol can generate angioblasts *via* mesodermal intermediates and the cells demonstrate dual potential to differentiate into haematopoietic and vascular lineages [21]. To direct angioblast differentiation towards functional EPCs, we tested whether endothelial growth media for mature endothelial cultures could specify and drive the angioblasts into EPCs more efficiently. In this study, we chose two media, EGM-2 and ECGS, and continued to culture angioblasts in ADM as a reference control. EGM-2 contains defined growth factors, whereas ECGS contains bovine pituitary extracts. After angioblast differentiation, we transferred EBs onto collagen IV-coated plates and cultured them for 12 days. Figure 2 illustrates the dynamic process of angioblast specifications.

To determine the proliferative potential of single cells, we first isolated them and then cultured them in the three media; at 15 days we counted the total cell number per colony from 20 colonies [Fig. 2A (a–c)]. Because we counted only attached cells, the average numbers per colony from EGM-2 and ECGS cultures were significantly higher than from ADM cultures [Fig. 2A (d)]. This result signified that ADM did not contain the essential factors for endothelial progenitor development towards anchorage-dependent cell lines. There was no statistical difference between cultures specified by EGM-2 and ECGS.

We then used flow cytometry to analyse the antigenic characteristics on the cell surface during specification under the three media. Figure 2B shows two-parameter histograms of multiple markers expressed in HPP-ECFCs (a); in angioblasts specified in ADM (b); in EGM-2 (c); and in ECGS (d). Several important changes occurred: the efficiencies of CD34+ production were different; the percentage of CD34+ cells in angioblasts specified in EGM-2 was 6.41 ± 1.16%; specified in ECGS was 9.32 ± 3.73%; and specified in ADM was 3.40 ± 0.92%, while HPP-ECFCs exhibited 97.70 ± 2.91% of cells as CD34+. However, angioblasts showed high CXCR4 positivity: 80.53 ± 8.25% under ADM, 58.41 ± 5.23% under EGM-2; and 49.04 ± 5.12% under ECGS; HPP-ECFCs highly expressed CXCR4 (96.76 ± 1.92%). It should be noted that angioblasts retained relatively higher UEA-1 in ADM (75.34 ± 5.61%). UEA-1 expression decreased under specified conditions (60.44 ± 4.45% in EGM-2; 49.95 ± 8.23% in ECGS). Its expression in HPP-ECFCs was 1.39 ± 2.07% and its expression in undifferentiated ESCs ranged from 85.56% to 92.07% (data not shown). In addition, HPP-ECFCs had few CXCR4+/UEA-1+ cells (0.74 ± 1.07%), but these cells made up 5.48 ± 2.11% in angioblasts cultured in ADM, 9.00 ± 2.05% in EGM-2 and 4.19 ± 1.25% in ECGS. Either HPP-ECFCs or specified angioblasts did not express CD31 and CD117. To further profile the antigenic characteristics in specified EPCs, we compared expression of a set of EPC or EC markers with adult HPP-ECFCs. As indicated in Figure 2C (a), 99.31% of HPP-ECFCs expressed CD34; 60.94% of CD34+ cells also stained positive for CXCR4 and the majority of HPP-ECFCs did not express CD146, VEGFR3, or CD62E. When angioblasts were specified in either EGM-2 [Fig. 2C (b)] or ECGS [Fig. 2C (c)], CD34+ cell generation was not significantly different, but angioblasts specified in ECGS had higher CD34+/CXCR4+ (2.95 ± 1.05%) than those specified in EGM-2 (0.49 ± 0.41%), which is a more similar phenotype to that of HPP-ECFCs. Angioblasts specified in either EGM-2 or ECGS did not express CD146, CD31, VEGFR3, CD62E or CD117.

### Development of endothelial functionalities

Furthermore, we demonstrated that EPCs generated from ESCs acquire functionalities specific to endothelial cells. Because functional endothelial cells are able to take up LDL *via* their receptors, we investigated whether specified angioblasts developed this functionality. As demonstrated in Figure 2D, angioblasts specified in ECGS (d) exhibited the highest uptake, followed by angioblasts specified in EGM-2 (c) or in ADM (b). We also examined the abilities of specified angioblasts to respond to TNF-α by measuring their cellular adhesion molecule expression (CD54, CD62E and CD106). As shown in Figure 2E, CD54 and CD106 responded significantly to TNF-α treatment in the four cell types, but CD62E expression was seen only in mature ECs. Although specified angioblasts exhibited lower responsiveness than mature ECs, TNF-α treatment resulted in up-regulation of certain cellular adhesion molecules in these cells, indicating that they developed the ability to respond to inflammatory cytokines. In addition, specified angioblasts and angioblasts in ADM also expressed CD54 at baseline, similarly to mature ECs. When we seeded specified angioblasts either in EGM-2 or in ECGS in Matrigel substrate, we saw a significant number of well-organized vascular structures, as shown in Figure 2F (a) shows how mature ECs seeded on Matrigel lined up and formed networks in 2 days. In contrast, specified angioblasts in EGM-2 [Fig. 2F (b and e)] and ECGS [Fig. 2F (c and f)] proliferated to a much greater extent than ECs during our observation period as a consequence of their stemness, and they developed 3D structures on the gel. More importantly, a portion of specified angioblasts could form microvessels with intact lumen structures in EGM-2 [Fig. 2F (b and e)], as shown by the red arrows; this vascular structure became more complex when angioblasts were specified in ECGS as demonstrated by multilayered, networked structures [Fig. 2F (c and f)]. However, when angioblasts grown in ADM were seeded on Matrigel, they showed sprouting after 2–3 days in culture [Fig. 2F (d)], but they failed to grow, indicating that ADM does not support cell growth or specification to functionally mature endothelial cells. The cells do not proliferate in ADM and disappear eventually. The small rounded objects are the remains of dying cells. We concluded that, being a heterogeneous mixture, angioblasts specified in EGM-2 and ECG contain a population(s) that possesses considerably higher vasculogenic potential than those specified in ADM, and that they function as endothelial progenitors.

### Maturation and reparative activity of CD34+ EPCs

**Fig. 3:**
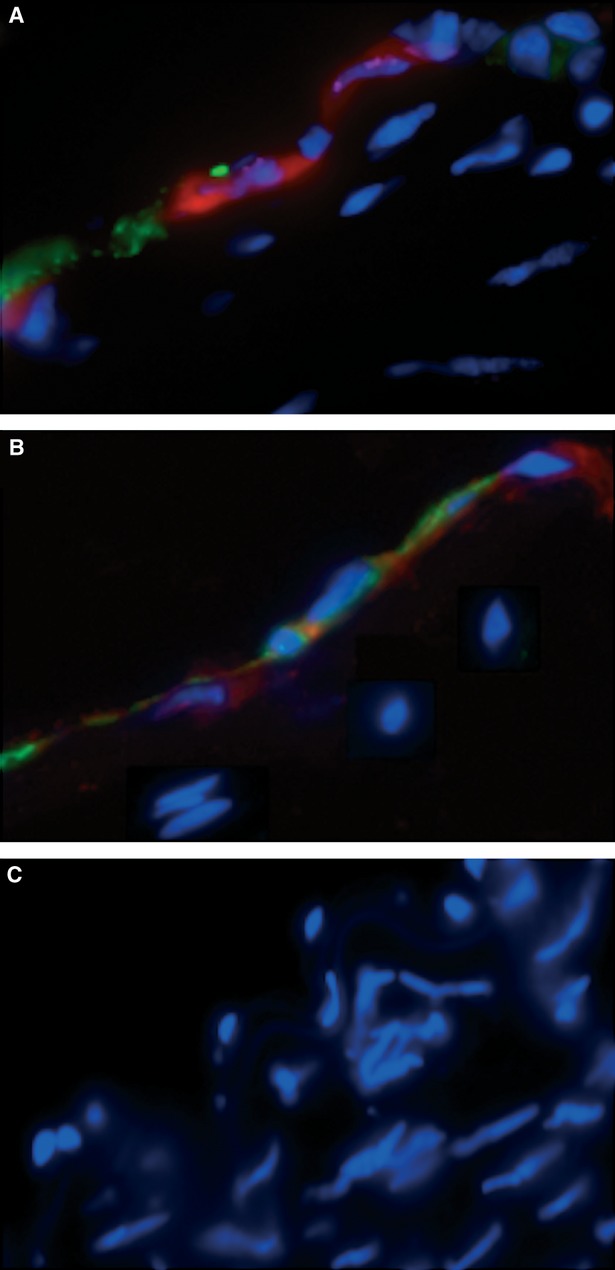
Endotheliazation of CD34+ EPCs derived from ESCs in *ex vivo* culture system. Attachment, growth and maturation of CD34+ EPCs on denuded artery surface. Transplanted cells labelled with CMRA attach, grow and mature on the interior surface of denuded blood vessels. The lumen of the vessel is at the upper left. (**A**) Transplanted cells express CD31 as demonstrated by anti-CD31 conjugated with FITC (green); (**B**) transplanted cells express CD146 as demonstrated by anti-CD146 conjugated with FITC (green); (**C**) is a vehicle control. Nuclei are stained blue with DAPI.

To test whether specified angioblasts could function as progenitors to execute a reparative role, we examined their ability to re-endothelialize blood vessels whose endothelium was removed by enzymatic digestion. We tested our hypothesis in an *ex vivo* bioreactor that we constructed. After culturing for approximately 2 weeks, a 0.5–0.7 cm segment was fixed, embedded in OCT and sectioned longitudinally into 10-μm-thick serial sections; whole-mount immunostaining was performed to assess the fate of the transplanted cells. Figure 3 shows cellular images acquired from serial sections of a representative *ex vivo* sample. Immunofluorescence staining indicated that transplanted CD34+ EPCs not only homed to and recovered the denuded surface but they also matured to fully differentiated cells by expressing CD31 (a) and CD146 (b), which were not expressed in angioblast-derived cells before seeding. This result implies that extracellular matrix of denuded vasculature possesses signalling molecules that direct and promote the transplanted CD34+ EPCs to gain full functional competence.

## Discussion

Although the *in vitro* differentiation of embryonic stem cells has proved to be an invaluable resource for producing large quantities of early progenitor cells, generating clinically useful cells remains a great challenge. The current investigation aims to establish a new approach to harvesting functional EPCs from ESCs-derived angioblasts for cell therapy by recapitulating their *in vivo* microenvironments from a developmental biology perspective. During vasculogenesis in the early embryo, mesodermal cells derive from the primitive streak and differentiate into endothelial precursors, known as angioblasts, as a result of migration and congregation of mesodermal stem cells [33, 34]. This represents a complex sequence of events regulated through the interplay of multiple cellular interactions [35]. Based on this concept, we have been suggested that it would be more efficient to generate progenitor cells under microenvironments analogous to those that exist during endothelial development *in vivo*. To test this hypothesis, we differentiated ESCs in a spatially and temporally dependent manner as occurs *in vivo*; we directed their differentiation through definitive mesoderm to angioblasts; and finally we specified them into progenitors (Fig. 1). Using this approach, we were able to improve the efficiency of functional EPC production and the efficacy of the derived EPCs.

Our approach has several important implications. In contrast with traditional one-step differentiation, our step-wise process has improved efficiency of generating CD34+ cells from ESCs. When we differentiated ESCs using the EB method in the presence of haematopoietic and endothelial growth factors [36–39], the CD34+ EPCs ranged within 0.27–3.39%; but when we used the step-wise differentiation protocol, the CD34+ EPCs increased to 5.25–13.05%. Although Woods *et al*. demonstrated that the production of CD34+ endothelial progenitor cells can be more efficient than that [40], many inhibitors and activators were used in that study and their effects on overall cellular activities have not been determined. Our approach relies on direct derivation from known and defined factors without interruption of existing cellular signalling pathways; therefore, it reflects the natural development of the progenitors.

In this study, we observed that the colony size of specified EPCs reached a plateau after 15 days in culture [Fig. 2A (a–c)], indicating that EPCs by our method have controlled and limited proliferation ability under culture conditions. The step-wise protocol allows us to progressively derive the primitive stem cells towards lineage-restricted differentiation; the cells gain more functionality and lose their proliferative potential while differentiating. This is critically important because finding the optimal maturation stage that retains an appropriate level of proliferative and regenerative potential for endothelium replacement is the priority for their therapeutic use. We anticipate that a well-differentiated cell source with decreased cellular plasticity will lower the possibility of teratoma formation, the foremost safety concern in stem cell therapy.

The CD34+ EPCs produced by this protocol were equipped with molecules that are essential for EPC to function as a reparative cell type. As shown in Figure 2B and C, some of the CD34+ EPCs coexpressed CXCR4 after specification, thus manifesting a similar expression pattern to HPP-ECFCs, which are putative tissue-resident progenitor cells. Coexpression of CD34 and CXCR4 is highly significant for EPCs to execute their biological function. Chen *et al*. suggested that CXCR4 plays a critical role in regulating initial vessel formation and functions as a morphogen during human embryonic vascular development [38]. The SDF-1/CXCR4 axis is the major transmembrane receptor that regulates the traffic of stem cells [41]. Because a subset of EPCs specified either by EGM-2 or ECGS formed complete, well-organized lumen structures or highly structured capillary networks [Fig. 2F (b, c, e and f)], we speculate that it was the cells with the CXCR4 signalling apparatus that modified the formation of capillary-like structures of ESC-derived endothelial cells. Further studies must be carried out to confirm this interpretation. In addition, specified EPCs possess basal expression of CD54 and CD106 and are able to react to inflammatory cytokines, to a greater or lesser extent, as shown in Figure 2E. CD54 and CD106, IgG superfamily members, are endothelial adhesion molecules that are involved in vascular integrity and inflammation; thus, it is important that endothelium derived from embryonic sources express these molecules because the ability to respond to circulating toxins or bacteria is an essential function of vasculature [36]. Other researchers have also pointed out that only EPCs with IgG superfamily proteins are able to recruit for ischaemic tissues by providing receptor targets for β-integrin and β_2_-integrin as demonstrated in recovery of hind-limb blood flow in experimentally ischaemic animals [42]. This evidence again indicates the advantage of the step-wise differentiation procedure.

To further determine the functionality of ESC-derived EPCs, we investigated whether specified cells have an immediate role in repairing the damaged vascular wall. We chose CD34+ cells because their expression changed significantly during derivation, and CD34 expression is considered as an important marker for circulating EPCs [9]. When they were transplanted, fluorescently labelled specified EPCs engrafted onto the surface of the denuded artery segment and re-endothelialized it to a high extent. We observed that transplanted cells recovered 60–70% of the denuded surface within 2 weeks after transplantation. Further study of the cross-section using immunohistochemical staining indicated that the transplanted cells underwent further maturation by expressing CD31 and CD146, which did not exist before seeding (Fig. 3A and B). This observation demonstrated that these CD34+ EPCs not only recover the injured artery surface but also integrate into the endothelium and regenerate the lost function. CD31 and CD146 are key cell surface proteins that constitute intercellular connections or junctions between cells and control the permeability of the endothelium, thus contributing to the endothelial barrier function. We believe that extracellular matrix materials from denuded vasculature may provide some instructive signals or niches to induce grafted EPCs to fully differentiate into functioning endothelial cells.

Our results strongly imply that angioblasts by themselves may not have a therapeutic effect; they need an additional specification stage to undergo lineage-restricted development and an appropriate microenvironment to mature as terminally functional cells. Although ESCs can be differentiated into the endothelial lineage, the identities and functionalities of the differentiated cells have been poorly defined; consequently, their effectiveness as therapeutic agents is in doubt [35, 36, 39, 42]. Our data suggest that angioblasts, after culturing in ADM, developed some endothelial functionalities such as LDL uptake and response to inflammatory cytokines (Fig. 2D and E); but they did not develop capacity for persistent anchorage-dependent proliferation or vascular structure formation (Fig. 2A and F). However, greater endothelial-specific functionalities developed when angioblasts were specified in media containing growth factors in environments appropriate for the well-developed cells, in keeping with the earlier suggestion that shear stress and inflammatory stimuli can facilitate the ability of ESCs to progress towards a more mature EC phenotype [36, 43]. The most provocative observation from our study was that a subset of EPCs specified either by EGM-2 or ECGS, when seeded on Matrigel, formed an entire vascular cavity or closed microvessels, indicating strong vascular regeneration ability. Even more striking, we saw a distinct difference between adult ECs derived from vasculature and EPCs derived from stem cells. ECs from adult vascular walls appear to be lined up and form cord-like structures on Matrigel [Fig. 2F (a)], but specified EPCs derived from ESCs can form completely vascularized structures including lumen [Fig. 2F (b and e)] or complex three-dimensional vasculature [Fig. 2F (c and f)]. We attributed this morphological difference to the origin of the cell source; HPP-ECFCs were isolated from adult tissue and displayed more angiogenic behaviours (the growth of new blood vessels from pre-existing vessels), but EPCs from ESCs underwent a vasculogenic process (the formation of new blood vessels when there is none that pre-exists). This means that EPCs generated in this protocol have acquired all of the molecules required for the morphogenic process and the intercellular communication that regulates this process. Specified ESC-derived EPCs have higher rejuvenating ability to form new blood vessels than cells from adult tissue.

Our results highlight not only a novel way to generate functional EPCs with high yield but also the value of using a non-human primate model for evaluating the therapeutic effects of stem cells. Animal modelling of human diseases has remained a critical challenge in stem cell-based therapy. We generated baboon EPCs with similar characteristics, such as antigenic markers, differentiation kinetics and functionalities, to those of human ESC-derived EPCs described in previous reports [37, 39]. Because of the high degree of physiological and genetic similarity between baboons and human beings, our results will have high relevance to human beings. The baboon model can more highly inform us about the biology of stem cells, especially the mechanisms that regulate their differentiation, which will be directly transplantable to regenerative and translational medicine in human beings.

Moreover, there has been no convincing evidence that confirms the therapeutic efficacy of engrafted stem cell derivatives in either human beings or animals for treating arterial damage. Using a denuded blood vessel, we created a disease model and demonstrated that the transplanted cells do have reparative effects, showing clear evidence of therapeutic potential. This bioassay can be extended to evaluate the functionalities of a variety of ESC-derived cells and to identify clinically useful cell types. It can also be a platform to study the causal mechanisms and the mode of action for stem cell derivatives. Because 50% of clinical trails of cell therapeutics are conducted without an understanding of their mode of action [44], our bioassay using a live tissue can bridge this gap.

## Supporting information

**Fig. S1** Characteristics of pluripotent baboon ESCs.

**Fig. S2** (**A**) Angioblast formation from EBs under ADM culturing. (**B**) Angioblast formation from EBs under ADM culturing. (**C**) Angioblast formation from EBs under ADM culturing. (**D**) Angioblast formation from EBs during ADM culturing. (**E**) Angioblast formation from EBs under ADM culturing.
