# Supplementary material for: Endothelial reconstitution by CD34+ progenitors derived from baboon embryonic stem cells
Source: J Cell Mol Med. 2013 Jan 10;17(2):242–51. doi: 10.1111/jcmm.12002 (PMC3814022; doi:10.1111/jcmm.12002)
Supplement: Supplementary file 1 [file jcmm0017-0242-SD1.pdf]

Figure 1. Characteristics of pluripotent baboon ESCs

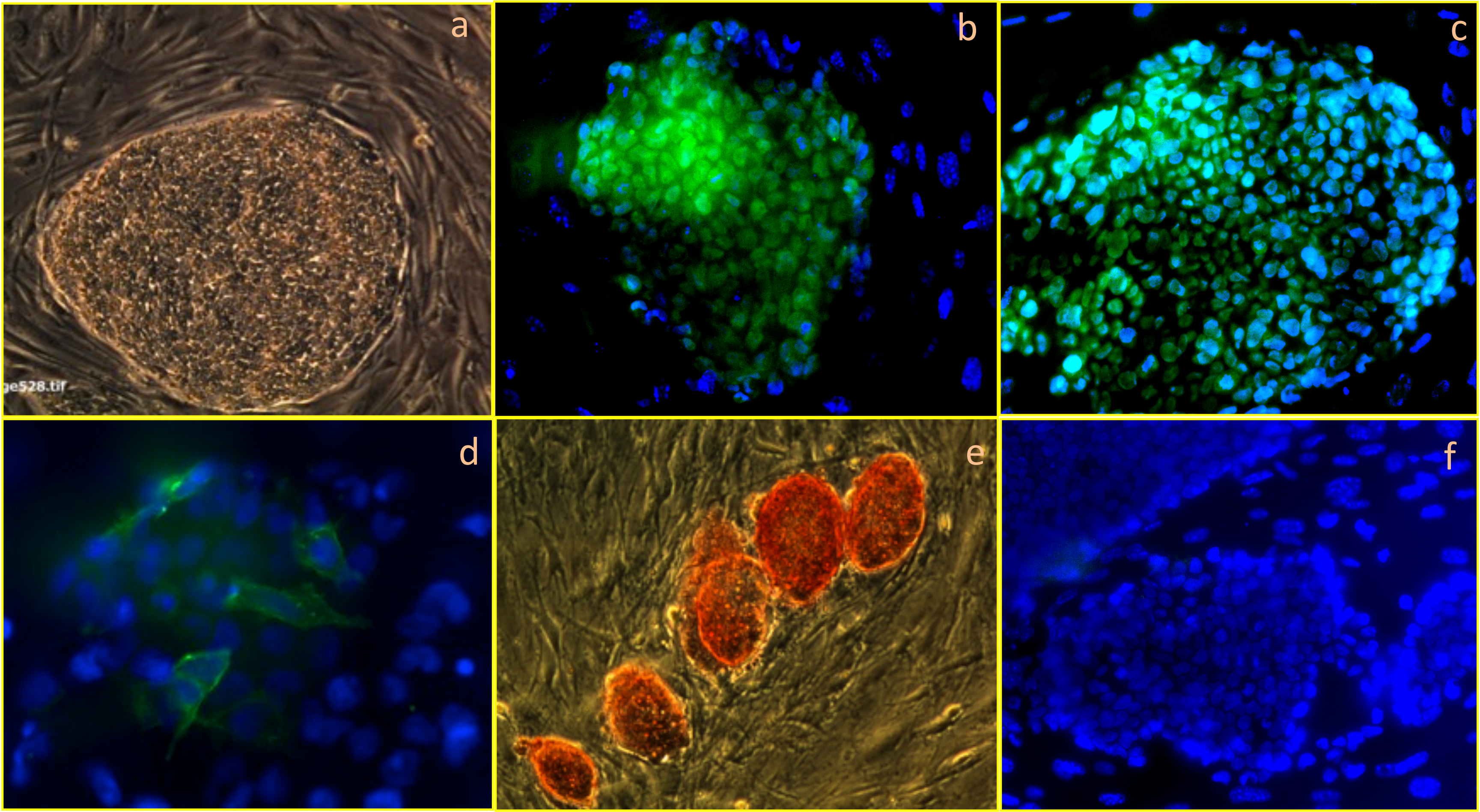

Pluripotency of baboon ESCs was confirmed by growth behavior in colonies (a, 100X) and positive immunostaining for NANOG (b, 200X), OCT-4 (c, 200X), SSEA-4 (d, 400X), and histochemical staining for ALP (e, 100X). Panel f was isotype image (f, 200X),

Figure 2A. Angioblast formation from EBs under ADM culturing

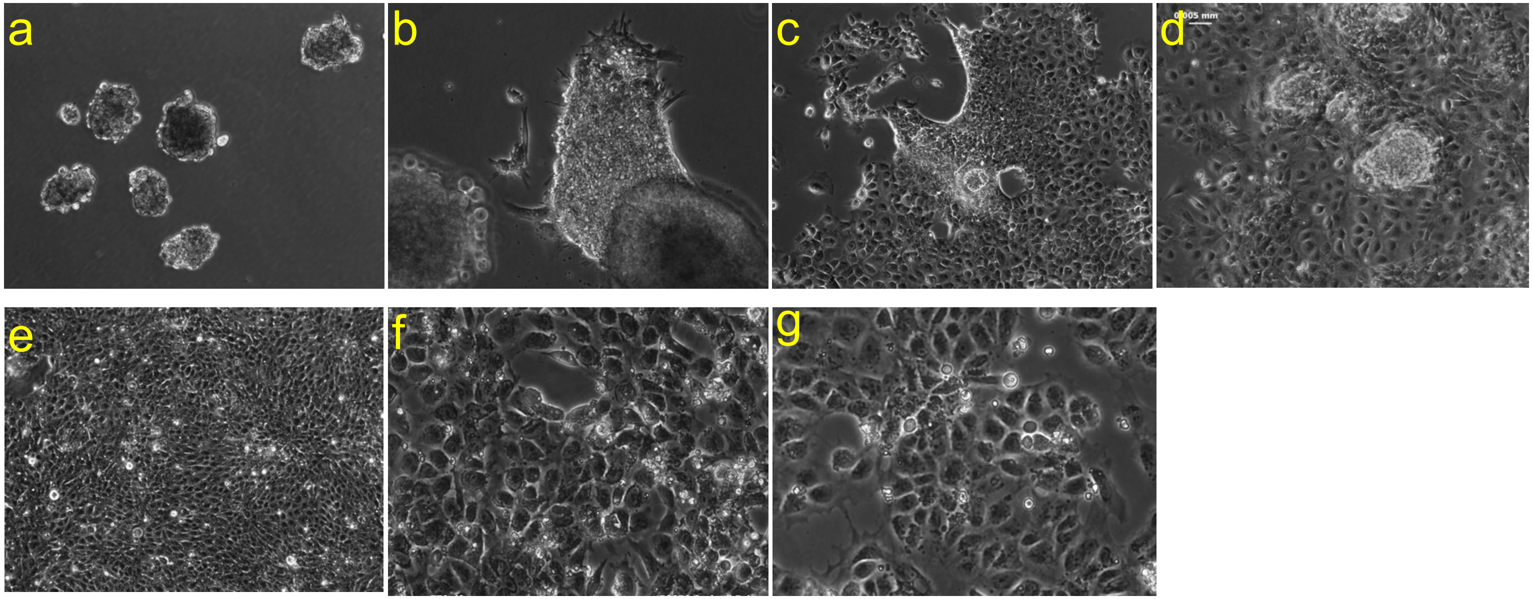

Morphological development of angioblasts under ADM culturing. Undifferentiated ESCs were induced to form EBs using AggreWell™ plates; 5,000–10,000 cells were contained in each EB (a). After 9 days of culture in ESC/ADM media at various ratios as indicated Figure 1, EBs were transferred onto collagen-coated plates and their morphological changes during differentiation were observed. Images b through g are representative of their growth features. One day after initiation of the monolayer culture (b), some cells grew out from EBs and continued to cover the plate after 3 days (c) and 5 days (d); the cultures became confluent after 9 days (e). Angioblast cultures appeared to have typical cobblestone morphology under phase-contrast microscopy from day 5 (f) and retained that morphology for three weeks (g). a-e, 100X. f-g 400X.

Figure 2B. Angioblast formation from EBs under ADM culturing

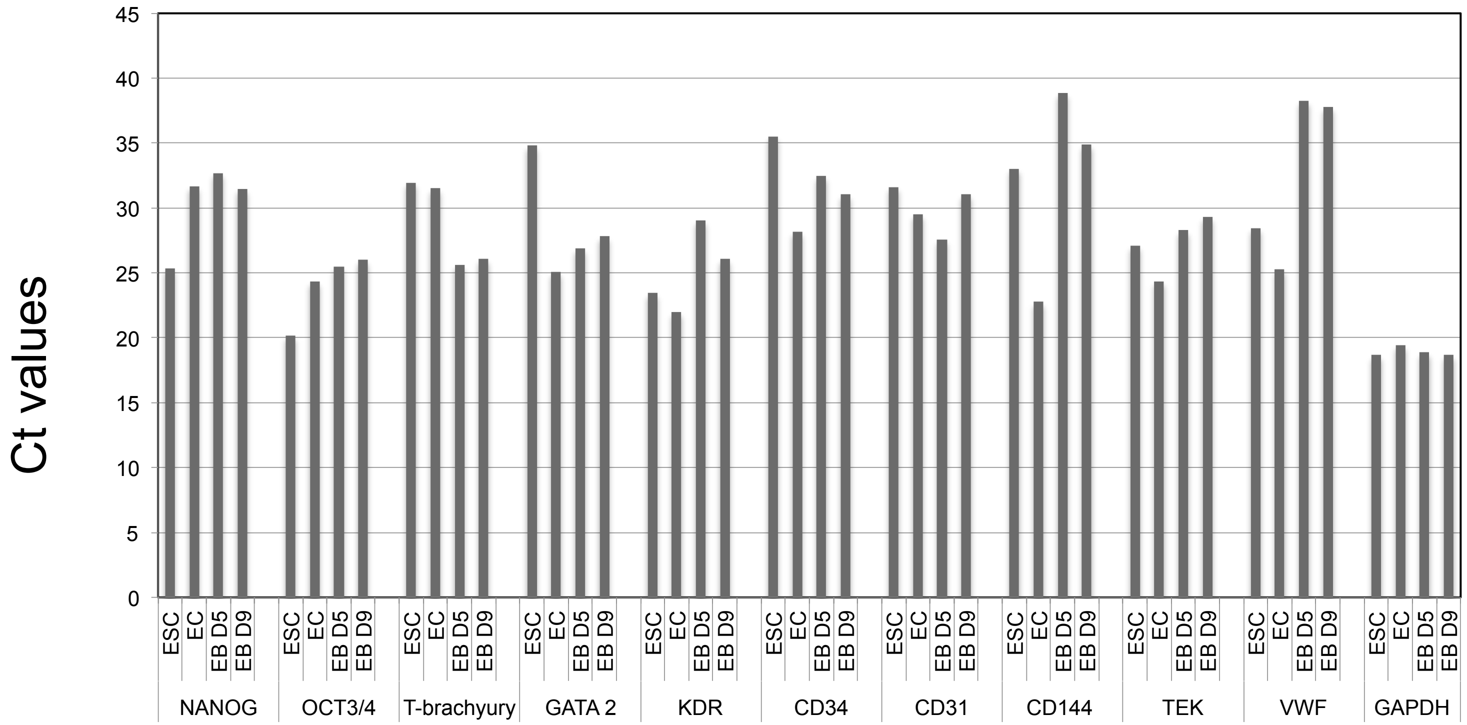

Gene expression analysis by RT-PCR of EB cultures grown in ADM for 5 and 9 days. Total RNA was isolated from ESC, EC and EB cultures at day 5 (D5) and day 9 (D9). Expression of 10 angioblast marker genes together with a housekeeping gene (GAPDH) was analyzed by quantitative real-time PCR. All mRNA expression levels are expressed as Ct values.

Figure 2C. Angioblast formation from EBs under ADM culturing

Immunohistochemical staining of mesodermal nuclear transcription factors T-brachyury and GATA2, and membrane KDR in ESCs (a, b, c) and embryoid bodies at day 5 (d, e, f). Panel g, isotype control. Nuclei are stained blue with DAPI. 400X

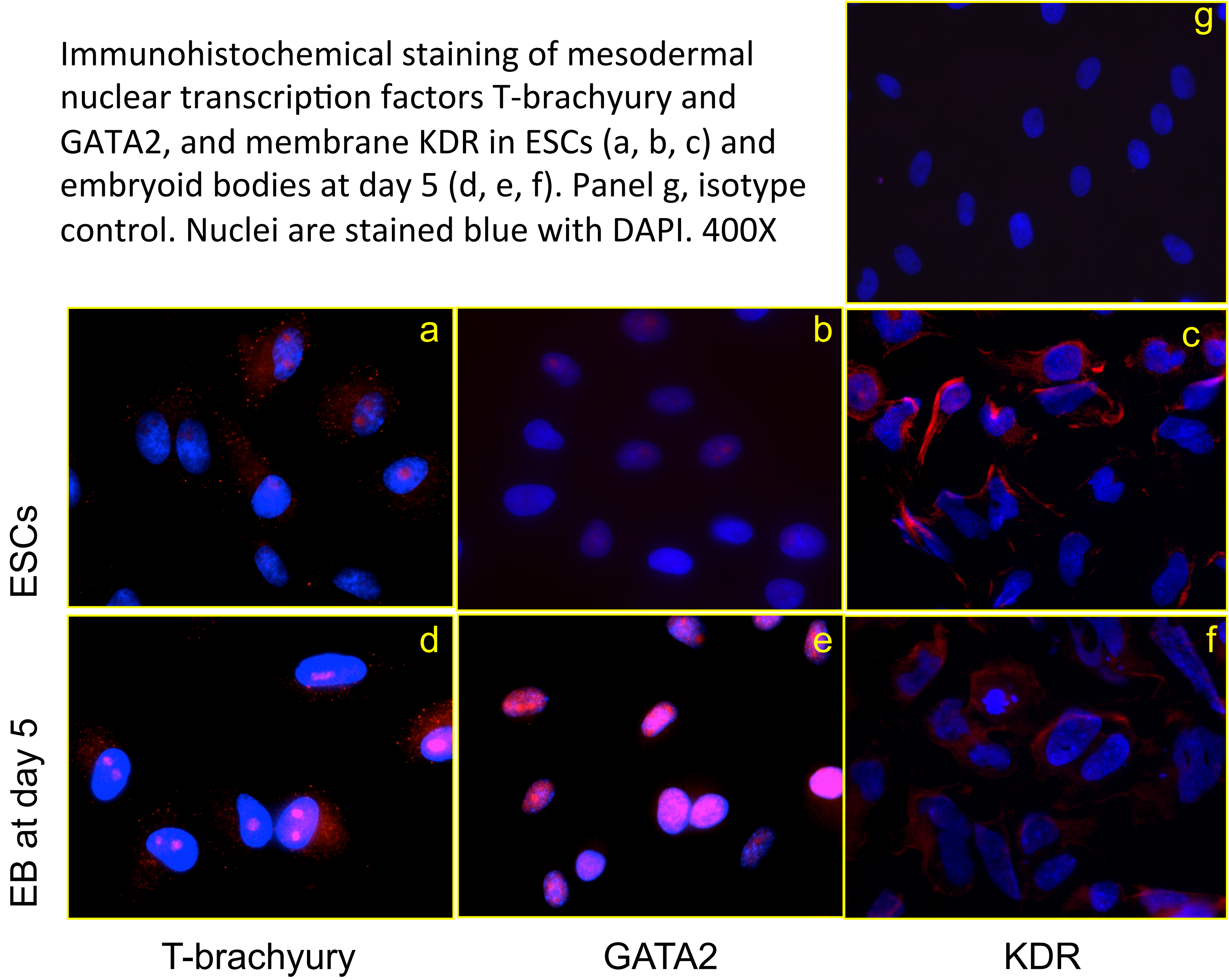

Figure 2D. Angioblast formation from EBs during ADM culturing

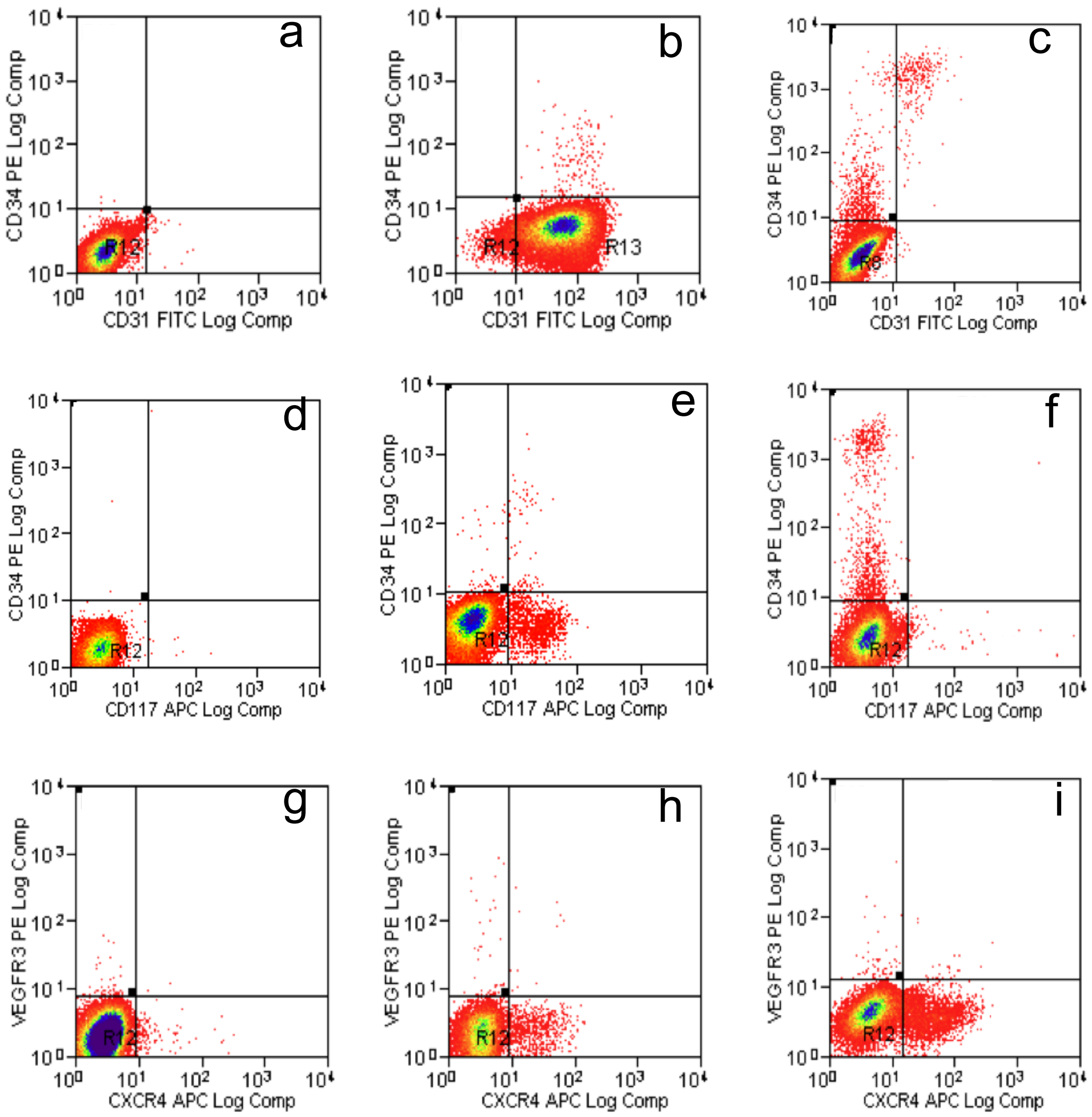

The kinetics of generation of angioblasts during culture of EBs in ADM. Flow cytometry results indicate the characteristics of phenotypic expression of several progenitor markers in pluripotent ESCs (a, d, g), in ESC-derived cells after 3 days differentiation (b, e, h), and in ESC-derived cells after 9 days differentiation (c, f, i).

Figure 2E. Angioblast formation from EBs under ADM culturing

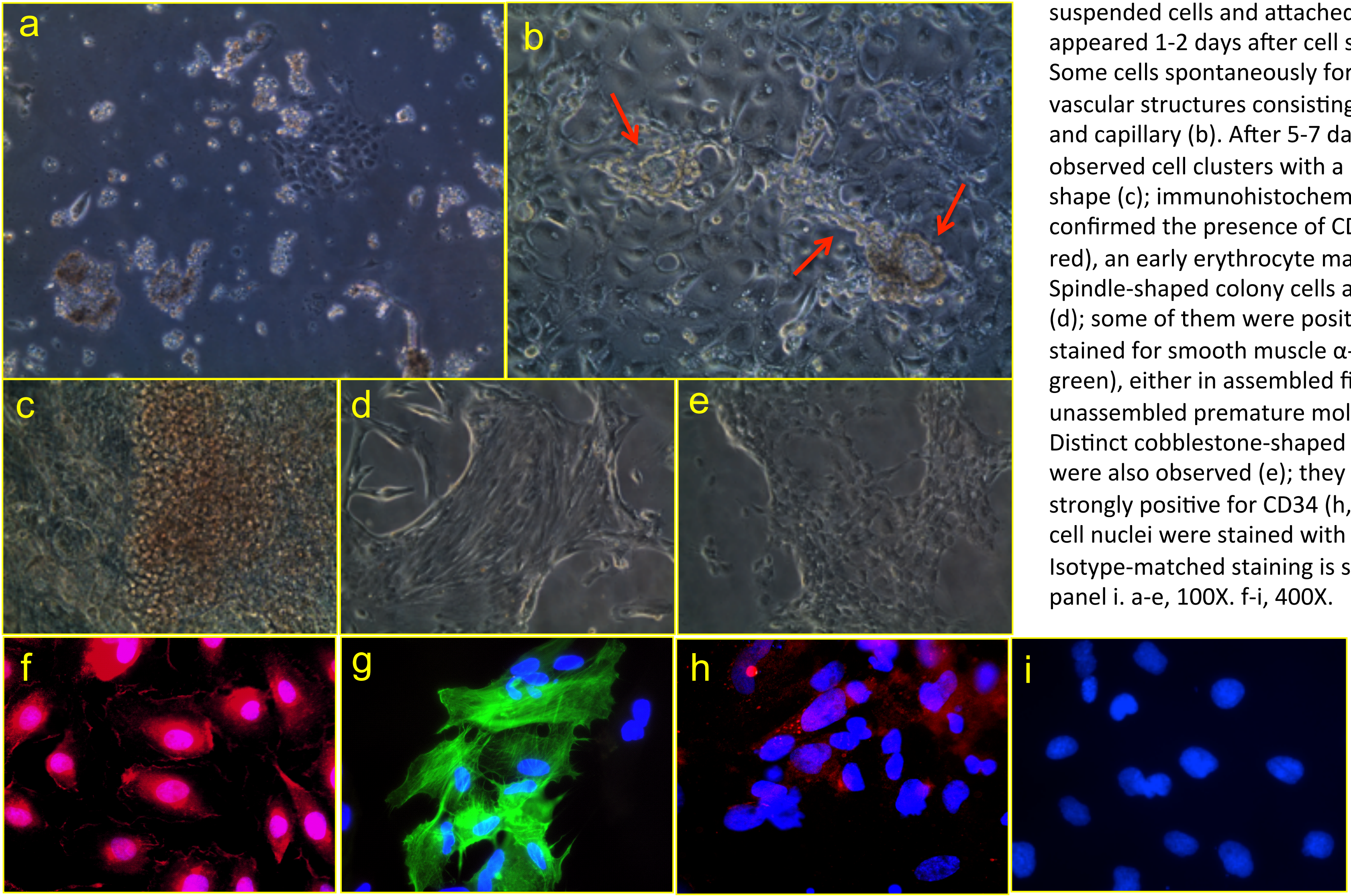

Dual differentiation of angioblasts toward hematopoietic and vascular lineages.

When angioblasts were cultured in monolayer, a mixed culture of suspended cells and attached cells (a) appeared 1-2 days after cell seeding. Some cells spontaneously formed vascular structures consisting of lumen and capillary (b). After 5-7 days, we observed cell clusters with a red grape-shape (c); immunohistochemical staining confirmed the presence of CD235a (f, red), an early erythrocyte marker. Spindle-shaped colony cells also existed (d); some of them were positively stained for smooth muscle α-actin (g, green), either in assembled fiber form or unassembled premature molecules. Distinct cobblestone-shaped cultures were also observed (e); they were strongly positive for CD34 (h, red). All cell nuclei were stained with DAPI (blue). Isotype-matched staining is shown in panel i. a-e, 100X. f-i, 400X.
